# Perivascular spaces relate to the course and cognition of Huntington’s disease

**DOI:** 10.1186/s40035-023-00359-9

**Published:** 2023-05-15

**Authors:** Xiao-Yan Li, Juan-Juan Xie, Jin-Hong Wang, Yu-Feng Bao, Yi Dong, Bin Gao, Ting Shen, Pei-Yu Huang, Hao-Chao Ying, Han Xu, Anna Wang Roe, Hsin-Yi Lai, Zhi-Ying Wu

**Affiliations:** 1 Department of Medical Genetics and Center for Rare Diseases, Department of Neurology in Second Affiliated Hospital, Key Laboratory of Medical Neurobiology of Zhejiang Province, Zhejiang University School of Medicine, Hangzhou, China; 2 Interdisciplinary Institute of Neuroscience and Technology, College of Biomedical Engineering and Instrument Science, Key Laboratory for Biomedical Engineering of Ministry of Education, Zhejiang University, Hangzhou, China; 3 College of Computer Science and Technology, Zhejiang University, Hangzhou, China; 4 Department of Radiology, Second Affiliated Hospital, Zhejiang University School of Medicine, Hangzhou, China; 5 School of Public Health, Zhejiang University, Hangzhou, China; 6 MOE Frontier Science Center for Brain Research and Brain-Machine Integration, School of Brain Science and Brain Medicine, Zhejiang University, Hangzhou, China; 7 CAS Center for Excellence in Brain Science and Intelligence Technology, Shanghai, China

Huntington’s disease (HD) is an autosomal dominant neurodegenerative disease that is caused by a cytosine-adenine-guanine (CAG) expansion in the first exon of the *huntingtin* (*HTT*) gene, which codes for the huntingtin protein. It typically manifests with a triad of symptoms, including motor disorders, cognitive impairment and psychiatric disturbances [1]. HD primarily affects the basal ganglia (BG), especially the caudate and putamen, after which it extends to more widespread gray and white matter [2]. Perivascular spaces (PVSs) are fluid-filled extensions of the subarachnoid spaces that enclose cerebral blood vessels and extend from the cerebral cortex through the brain parenchyma. The physiological role of PVSs is the drainage of brain interstitial fluid into perivascular pathways for the elimination of waste products through the glymphatic drainage system. An increasing number of studies have demonstrated that enlarged PVSs indicate glymphatic dysfunction and are associated with many neurological diseases, such as Alzheimer’s disease, Parkinson’s disease and small vessel disease [3]. With the advantage of strong field strengths, 7.0 T images show superior resolution and signal-to-noise ratios than 3.0 T, which facilitate the visualization of PVS. And automated segmentation methods could accurately identify PVS in a short time with no inter-rater variability. In the current study, we used U-shaped networks (U-net), a class of deep learning methods, to explore the PVS distribution in HD and controls. To date, PVS distribution in HD is still unclear. Only two studies have investigated PVSs in HD, and both demonstrated increased visible PVS burden in manifest HD compared to controls [4, 5]. However, whether PVS burden increases in premanifest HD (pre-HD) individuals remains unknown, and the relationship of PVS with cognitive decline has never been studied.

In this study, 49 healthy controls, 32 pre-HD individuals and 25 HD patients were enrolled and assessed by using 7.0 T MRI. Cognitive performance was assessed with a battery of cognitive tests, including Symbol Digit Modality Test, Stroop Word Reading Test, Stroop Color Naming Test, and Stroop Interference Test. U-net algorithm was used to automatically segment PVS with a diameter < 3 mm on T2-weighted images. Three metrics were computed to assess the performance of segmentation on the training set and validation set: Dice similarity coefficient (DSC), sensitivity (SEN) and positive prediction value (PPV). PVS volume proportion (%) was calculated as the regional PVS volumes over the total regional volumes. Detailed methods are provided in Additional file 1: Supplementary Methods.

There was no difference in sex ratio among control, pre-HD and HD groups (*P* = 0.520). The HD group had a significantly older age than the pre-HD and control groups (*F* = 24.03, *P* < 0.0001; *F* = 12.09, *P* = 0.001, Bonferroni-corrected threshold = 0.017). However, there was no difference in age between the control and the pre-HD groups (*F* = 0.250, *P* = 0.619). Detailed demographic and clinical features of the participants are shown in Additional file 1: Table S1. In the training subset of 30 subjects, the average value of DSC was 0.85, and the PPV and SEN values were 0.94 and 0.78, respectively. In the validation data, the DSC was 0.76, and the PPV and SEN values were 0.89 and 0.67, respectively. The performance of the automatic segmentation of PVS was generally good, and examples of PVS segmentation are shown in Fig. 1a–b. We then assessed the association of PVS volume with age. Global-brain PVS volume proportion (global-pPVS) increased with age in the controls (*r* = 0.35, *P* = 0.010) but not in *HTT* mutation-carriers (*r* = − 0.11, *P* = 0.496). However, the BG PVS volume proportion (BG-pPVS) increased with age in both controls (*r* = 0.63, *P* < 0.0001) and *HTT* mutation-carriers (*r* = 0.43, *P* = 0.007, Fig. 1c, d). The BG-pPVS differed among the pre-HD, manifest HD and control groups (*F* = 54.72, *P* < 0.0001) while the global-pPVS did not (*F* = 1.87, *P* = 0.159). After age adjustment, pre-HD and HD individuals had higher BG-pPVS than controls (*F* = 17.64, *P* = 0.001; *F* = 53.62, *P* < 0.0001). In addition, the HD group had higher BG-pPVS than pre-HD (*F* = 19.11, *P* < 0.0001, Bonferroni-corrected threshold = 0.017, Fig. 1e–f). By using partial correlation analysis to control for the age effect, we found that the BG-pPVS was negatively associated with the putamen volume (*r* = − 0.32, *P* = 0.036) in controls (Additional file 1: Table S2). In *HTT* mutation-carriers, BG-pPVS was inversely associated with both caudate volume (*r* = − 0.51, *P* < 0.0001) and putamen volume (*r* = − 0.39,* P* = 0.013) (Fig. 1g, h). In contrast, no associations of global-pPVS with brain atrophy were detected. We then explored the relationship of PVS burden with cognitive measures. Consistent with our hypothesis, in *HTT* mutation-carriers, BG-pPVS was negatively correlated with cognitive scores on the Stroop Word Reading Test (*r* = − 0.35, *P* = 0.010) and Symbol Digit Modality Test (*r* = − 0.39, *P* = 0.009) after age adjustments (Fig. 1i, j and Additional file 1: Table S2).

**Fig. 1:**
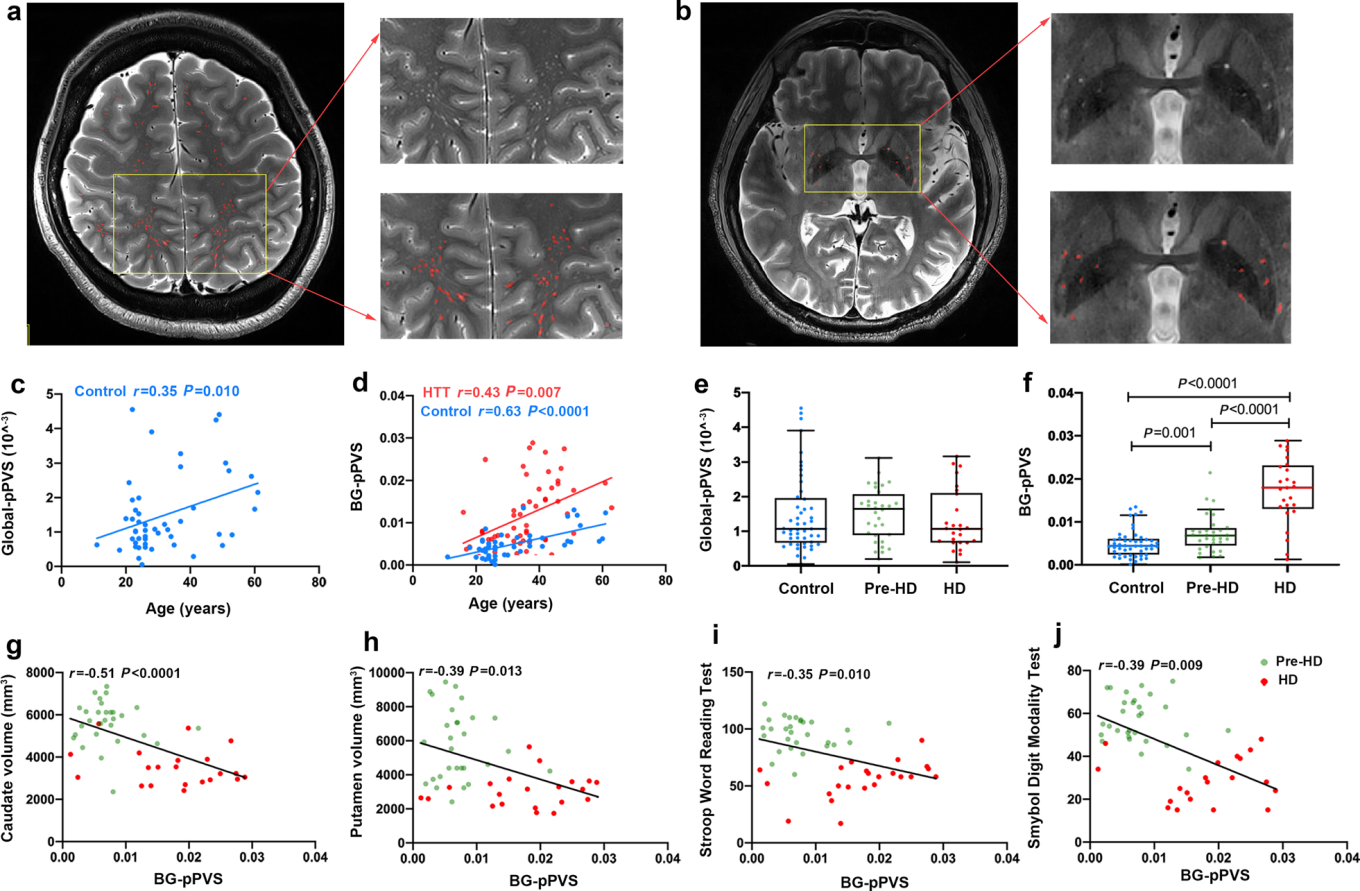
Perivascular space (PVS) distribution in *HTT* mutation-carriers and its associations with brain atrophy and cognitive decline. **a**, **b** Performance of automatic segmentation of PVS on T2-weighted axial images in the deep white matter and basal ganglia (BG). **c**, **d** Correlations of global-brain PVS volume proportion (global-pPVS) and BG PVS volume proportion (BG-pPVS) with age in controls and *HTT* mutation-carriers. **e**, **f** Group comparisons of global-pPVS and BG-pPVS between control, pre-HD and HD groups. Box plots show median and interquartile range (IQR), and whiskers are data within 1.5 IQR of the median. A multiple linear regression was used for group comparison to control for the effect of age. **g**, **h** Correlations of BG-pPVS with caudate and putamen volumes after age adjustment. **i**, **j** Correlations of BG-pPVS with Stroop Word Reading Test and Symbol Digit Modality Test scores after age adjustment

In this study, with the advanced segmentation algorithm and high-resolution 7.0 T MRI to quantify PVS burden, we found significantly increased BG-pPVS not only in HD patients but also in pre-HD individuals compared to controls. We also found that BG-pPVS was closely related to cognitive decline and BG atrophy. The performance of our automatic segmentation algorithm was comparable to that reported in other studies. Lian et al. [6] used a multi-scale encoder-decoder network on 7.0 T T2-weighted images to annotate PVS, and reported a performance of DSC 0.77, PPV 0.83 and SEN 0.74 at the voxel level. Zhang et al. [7] used a structured-learning-based segmentation framework to segment PVS on 7.0 T T2-weighted images and reported a DSC coefficient of 0.66 at the voxel level. Boutinaud et al. reported DSC 0.73 and SEN 0.71 for BG-PVS at the cluster level on 3.0 T T1-weighted images [8].

There were also some limitations in this study. First, the cognitive measures we used were mainly focused on the executive domain of cognition. Other cognitive tests assessing different cognitive domains should be used, such as the Cambridge Neuropsychological Test Automated Battery (CANTAB) Intra-Extra Dimensional Set-Shift (IED) task, which could detect mild cognitive impairments in pre-HD individuals far from onset and measure cognitive flexibility [9, 10]. Second, HD patients at the late disease stage were not included in this study, as they are unable to undergo MRI scanning due to obvious involuntary movements. Third, long-term follow-ups for imaging and cognitive measures are needed to determine the clinical relevance of PVS and the risk of dementia.

In summary, the current study shows that BG-pPVS increases in pre-HD individuals and is associated with early cognitive impairment and brain atrophy in HD. Diffusion tensor image analysis along the perivascular space should be carried out to accurately evaluate the glymphatic function in the future. Strategies to improve the glymphatic function may restore the cognitive impairment of HD patients and facilitate the delivery of intrathecal drugs.

## Supplementary Information

**Additional file 1.** **Supplementary Methods. Table S1.** Demographic and clinical characteristics of participants in the study. **Table S2.** Correlations of PVS volumes with imaging and clinical measures after age adjustment.

## Data Availability

The data presented in this study are available from the corresponding author on reasonable request.

## Abbreviations

HD: Huntington’s disease
BG: Basal ganglia
PVS: Perivascular spaces
DSC: Dice similarity coefficient
SEN: Sensitivity
PPV: Positive prediction value
