# Supplementary material for: Perivascular spaces relate to the course and cognition of Huntington’s disease
Source: Transl Neurodegener. 2023 May 15;12:30. doi: 10.1186/s40035-023-00359-9 (PMC10245407; doi:10.1186/s40035-023-00359-9)
Supplement: Supplementary file 1 — Additional file 1. Supplementary Methods. Table S1. Demographic and clinical characteristics of participants in the study. Table S2. Correlations of PVS volumes with imaging and clinical measures after age adjustment. [file 40035_2023_359_MOESM1_ESM.docx]

**Additional file 1**

**Perivascular spaces relate to the course and cognition of Huntington’s disease**

Xiao-Yan Li^1#^, Juan-Juan Xie^1, 2#^, Jin-Hong Wang^3^, Yu-Feng Bao^1^, Yi Dong^1^, Bin Gao^1^, Ting Shen^2^, Pei-Yu Huang^4^, Hao-Chao Ying^5^, Han Xu^1, 6^, Anna Wang Roe^1, 2, 6*^, Hsin-Yi Lai^1, 2, 6*^, Zhi-Ying Wu^1,6,7,8*^

^1^Department of Medical Genetics and Center for Rare Diseases, and Department of Neurology in Second Affiliated Hospital, and Key Laboratory of Medical Neurobiology of Zhejiang Province, Zhejiang University School of Medicine, Hangzhou, China;

^2^Interdisciplinary Institute of Neuroscience and Technology, and College of Biomedical Engineering and Instrument Science, Key Laboratory for Biomedical Engineering of Ministry of Education, Zhejiang University, Hangzhou, China;

^3^College of Computer Science and Technology, Zhejiang University, Hangzhou, China;

^4^Department of Radiology, Second Affiliated Hospital, Zhejiang University School of Medicine, Hangzhou, China;

^5^School of Public Health, Zhejiang University, Hangzhou, China;

^6^MOE Frontier Science Center for Brain Research and Brain-Machine Integration, School of Brain Science and Brain Medicine, Zhejiang University, Hangzhou, China;

^7^CAS Center for Excellence in Brain Science and Intelligence Technology, Shanghai, China.

^8^Lead contact.

**Supplementary Methods**

**Participants and study design**

In this study, 32 pre-HD individuals and 25 HD patients were recruited from the Second Affiliated Hospital of Zhejiang University School of Medicine, and 49 healthy controls without neurological diseases were recruited from the nearby communities between December 2017 and April 2022. Both pre-HD and HD were assessed via the Chinese version of Unified Huntington's Disease Rating Scale (UHDRS) [1]. The pre-HD individual was enrolled by using a positive *HTT* genetic testing and a total motor score of 5 or less in the UHDRS [2]. The manifest HD patient was diagnosed by at least two senior neurologists based on the clinical manifestations in the context of a positive *HTT* genetic testing, as described in our previous study [3]. All of the participants were assessed by using 7.0 T MRI. The study was approved by the local ethics committee of the Second Affiliated Hospital, Zhejiang University School of Medicine. All of the participants signed written informed consent forms.

**Clinical measurements**

Cognitive performance was assessed with a battery of cognitive tests, which comprised the Symbol Digit Modality Test, Stroop Word Reading test, Stroop Color Naming test, Stroop Interference test.

**Brain MRI image acquisition and processing**

Brain MRI scanning was performed on a 7.0 T Magnetom research system (Siemens Healthcare, Erlangen, Germany) with prototype sequences including a magnetization prepared with two rapid gradient echo (MP2RAGE) sequences. The sequence of T1-weighted images were the followings: repetition time (TR) = 5,000 ms, inversion time (TI)1 = 900 ms, TI2 = 2,750 ms, echo time (TE) = 2.27 ms, voxel size = 0.7 × 0.7 × 0.7 mm^3^. And a turbo spin echo (TSE) sequence of T2-weighted images were the followings: TR = 7,000 ms, TE = 66 ms, voxel size = 0.5 × 0.5 × 2.4 mm^3^.

T1­weighted MP2RAGE images were further segmented by FreeSurfer software (<https://surfer.nmr.mgh.harvard.edu>) and automatically labeled with the Desikan-Killiany-Tourville atlas. Cortical thickness, putamen, caudate and globus pallidus volumes were further extracted from the atlas. For more accurate volumetric measures of gray matter and white matter [4], we used the Computational Anatomy Toolbox 12 (CAT12, http://dbm.neuro.uni-jena.de/cat/) via the Statistical Parametric Mapping 12 software (SPM12, http://[www.fil.ion.ucl.ac.uk/spm/software/ spm12](http://www.fil.ion.ucl.ac.uk/spm/software/%20spm12)).

**Automatic segment of PVS**

The T2-weighted images were used to quantify the volumes of PVS in the whole brain, as PVS on T2-weighted images are more visible than T1-weighted images [5]. The diameter of the PVS we studied is <3 mm. When considering that manual segmentation is time-consuming and varies according to raters, we used a U-shaped network (U-net) to explore the PVS distribution in HD. U-Net is a fully convolutional network (FCN) with an encoder-decoder architecture. Compared to traditional FCNs, U-Net strengthens the extraction of low-level features through skip connections in the process of hierarchical upsampling. Benefiting from the rich spatial information in the shallow features, U-Net can generate more refined segmentation masks. To train and test the performance of the U-net algorithm, we randomly selected 10 samples from each group (control, pre-HD and HD group) to constitute a subset of 30 sample as training and validation data, the remaining samples as testing set. Li XY performed a voxel-wise manual delineation of PVS on the T2-weighted images from a subset of 30 sample using ITK-SNAP software [6]. We validated our segmentation approach in the training/validation set by performing 5-fold cross-validation. Furthermore, we assessed its performance in the testing set. The input were the T2-weighted images, and the output were the manually annotated masks. Each fold contained 80% training (24 samples) and 20% (6 samples) validation. Three metrics were computed to assess the performance of segmentation algorithm on the training sets and validation set. The three metrics were Dice similarity coefficient (DSC), sensitivity (SEN) and positive prediction value (PPV). These were defined by true positive (TP), false positive (FP) and false negative (FN) terms. The formulas were represented as follows:

DSC=$\frac{2TP}{2TP+FP+FN}$; SEN=$\frac{\mathrm{TP}}{TP+FN}$; PPV=$\frac{\mathrm{TP}}{TP+FP}$.

According to the method reported previously [7], we defined a cluster-level standard to count the TP, FP and FN: Clusters were deﬁned by a 26-neighbor connectivity rule that one voxel was connected to its surrounding 26 voxels. TP is calculated by the rule that a cluster of the predicted PVS volume included at least one voxel of the manually annotated PVS volume. Similarly, FP is calculated by the rule that a cluster of the predicted PVS volume included no voxels of the manually annotated PVS volume. Conversely, FN is calculated by the rule that a cluster of the manually annotated PVS volume included no voxels of the predicted PVS volume.

For regional PVS evaluation, the PVS volume proportion (%) was calculated as the regional PVS volumes over the total regional volumes. For example, the global-brain PVS volume proportion (global-pPVS) was calculated as the whole-brain PVS volumes over the whole white and gray matter. Moreover, basal ganglia PVS volume proportion (BG-pPVS) was expressed as BG-PVS volumes over BG volumes. A BG mask was created for each sample, which included subcortical nuclei identified by FreeSurfer as caudate, putamen and pallidum regions.

**Statistical analysis**

Statistical analysis was performed with SPSS software (version 20, IBM Corporation, Armonk, NY, USA). The Shapiro‒Wilk test was used to test normality. Nonparametric Mann-Whitney U test was used to compare CAG repeat length between groups. A linear regression was used to compare age between groups. A multiple linear regression was performed to compare PVS volumes with age adjustment. Bonferroni correction test was used to correct the *P* value after multiple group comparisons. A Chi-Square test was used to compare sex distribution. A partial correlation analysis was performed to calculate the correlation coefficients between PVS volumes and clinical and imaging measures after controlling for age*.* In addition, *P* < 0.05 was considered to indicate statistical significance.

**References**

1. Li X Y, Y F Bao, J J Xie, S X Qian, B Gao, M Xu, et al. The Chinese version of UHDRS in Huntington's disease: reliability and validity assessment. J Huntingtons Dis. 2022.

2. Tabrizi S J, R Reilmann, R A Roos, A Durr, B Leavitt, G Owen, et al. Potential endpoints for clinical trials in premanifest and early Huntington's disease in the TRACK-HD study: analysis of 24 month observational data. Lancet Neurol. 2012; 11(1):42-53.

3. Dong Y, Y M Sun, Z J Liu, W Ni, S S Shi, and Z Y Wu. Chinese patients with Huntington's disease initially presenting with spinocerebellar ataxia. Clin Genet. 2013; 83(4):380-3.

4. Eggert L D, J Sommer, A Jansen, T Kircher, and C Konrad. Accuracy and reliability of automated gray matter segmentation pathways on real and simulated structural magnetic resonance images of the human brain. PLoS One. 2012; 7(9):e45081.

5. Hernandez Mdel C, R J Piper, X Wang, I J Deary, and J M Wardlaw. Towards the automatic computational assessment of enlarged perivascular spaces on brain magnetic resonance images: a systematic review. J Magn Reson Imaging. 2013; 38(4):774-85.

6. Yushkevich P A, J Piven, H C Hazlett, R G Smith, S Ho, J C Gee, et al. User-guided 3D active contour segmentation of anatomical structures: significantly improved efficiency and reliability. Neuroimage. 2006; 31(3):1116-28.

7. Boutinaud P, A Tsuchida, A Laurent, F Adonias, Z Hanifehlou, V Nozais, et al. 3D segmentation of perivascular spaces on T1-weighted 3 Tesla MR images with a convolutional autoencoder and a U-shaped neural network. Front Neuroinform. 2021; 15:641600.

**Supplementary Table**

**Table S1** Demographic and clinical characteristics of participants in the study

|  | Controls | Pre-HD | HD | P |
| --- | --- | --- | --- | --- |
| Number | 49 | 32 | 25 | / |
| Female, n (%) | 23 (46.9%) | 15 (46.8%) | 15 (60.0%) | 0.520 |
| Age, mean (SD), years | 32.1 (12.4) | 30.9 (7.5) | 42.0 (10.2) | <0.0001 |
| CAG repeats, mean (SD) | / | 43.1 (2.5) | 46.2 (4.4) | 0.001 |
| Onset age, mean (SD), years | / | / | 38.2 (9.9) | / |
| Duration, mean (SD), years | / | / | 3.9 (3.5) | / |

Abbreviations: HD, Huntington’s disease; pre-HD, premanifest HD.

**Table S2** Correlations of PVS volumes with imaging and clinical measures after age adjustment

| Correlation coefficients | Global-pPVS | BG-pPVS |
| --- | --- | --- |
| **Controls** |  |  |
| Gray matter | -0.07 | 0.07 |
| White matter | 0.09 | -0.13 |
| Cortical thickness | 0.15 | -0.03 |
| Caudate | -0.13 | -0.28 |
| Putamen | -0.03 | -0.32***** |
| Pallidus | -0.21 | -0.07 |
| ***HTT* mutation carriers** |  |  |
| Gray matter | 0.12 | -0.18 |
| White matter | 0.09 | -0.05 |
| Cortical thickness | 0.22 | 0.003 |
| Caudate | 0.03 | -0.51*** |
| Putamen | -0.03 | -0.39* |
| Pallidus | -0.001 | -0.11 |
| Stroop Word Reading test | 0.18 | -0.35* |
| Stroop Color Naming test | 0.25 | -0.08 |
| Stroop Interference test | 0.11 | -0.23 |
| Symbol Digit Modality Test | 0.07 | -0.39** |

Abbreviations: PVS, perivascular spaces; Global-pPVS, global-brain PVS volume proportion; BG-pPVS, basal ganglia PVS volume proportion. **P*<0.05, ***P*<0.01, ****P*<0.001.
